# Anti-Atherosclerotic Effect of a Polyphenol-Rich Ingredient, Oleactiv^®^, in a Hypercholesterolemia-Induced *Golden Syrian* Hamster Model

**DOI:** 10.3390/nu10101511

**Published:** 2018-10-15

**Authors:** Cindy Romain, Antonio Piemontese, Simone Battista, Franco Bernini, Alice Ossoli, Arianna Strazzella, Sylvie Gaillet, Jean-Max Rouanet, Julien Cases, Ilaria Zanotti

**Affiliations:** 1 Fytexia SAS, Innovation and Scientific Affairs, 34350 Vendres, France; cromain@fytexia.com; 2 Dipartimento di Scienze degli Alimenti e del Farmaco, Università degli Studi di Parma, 43124 Parma, Italy; antonio.piemontese88@gmail.com (A.P.); simone.battista91@gmail.com (S.B.); f.bernini@unipr.it (F.B.); ilaria.zanotti@unipr.it (I.Z.); 3 Centro E. Grossi Paoletti, Dipartimento di Scienze Farmacologiche e Biomolecolari, Università degli Studi di Milano, 20133 Milano, Italy; alice.ossoli@unimi.it (A.O.); arianna.strazzella@unimi.it (A.S.); 4 Nutrition & Métabolisme, UMR 204 NUTRIPASS, Prévention des Malnutritions & des Pathologies Associées, Université Montpellier Sud de France, 34394 Montpellier, France; sylvie.gaillet-foulon@univ-montp2.fr (S.G.); jeanmax.rouanet@gmail.com (J.-M.R.)

**Keywords:** atherosclerosis, cholesterol efflux capacity, reverse cholesterol transport, high fat diet, polyphenol, artichoke, olive, grape, aortic fatty streak, atheroma plaque

## Abstract

The development of nutraceutical ingredients has risen as a nutritional solution for health prevention. This study evaluated the effects of Oleactiv^®^, an ingredient developed for the prevention of atherogenesis, in hypercholesterolemic hamsters. Oleactiv^®^ is a polyphenol-rich ingredient obtained from artichoke, olive and grape extracts as part of fruit and vegetables commonly consumed within the Mediterranean diet. A total of 21 *Golden Syrian* hamsters were divided into three groups. The standard group (STD) was fed a normolipidemic diet for 12 weeks, while the control group (CTRL) and Oleactiv^®^ goup (OLE) were fed a high-fat diet. After sacrifice, the aortic fatty streak area (AFSA), plasmatic total cholesterol (TC), high-density lipoproteins (HDL-C), non-HDL-C and triglycerides (TG), were assessed. The cholesterol efflux capacity (CEC) of hamster plasma was quantified using a radiolabeled technique in murine macrophages J774. OLE administration induced a significant reduction of AFSA (−69%, *p* < 0.0001). Hamsters of the OLE group showed a significant decrease of both non-HDL-C (−173 mmol/L, *p* < 0.05) and TG (−154 mmol/L, *p* < 0.05). Interestingly, OLE induced a significant increase of total CEC (+17,33%, *p* < 0,05). Oleactiv^®^ supplementation prevented atheroma development and had positive effects on the lipid profile of hypercholesterolemic hamsters. The increased CEC underlines the anti-atherosclerotic mechanism at the root of the atheroma reduction observed.

## 1. Introduction

Cardiovascular diseases (CVDs) are one of the major causes of death in Western countries. A total of 17.5 million people die each year from CVDs, corresponding to an estimated rate of 31% of all deaths worldwide [1], and this could remain the case for at least the next 15–20 years [1]. The main cause of the development of CVDs, atherosclerosis, has a multifactorial, gradual, and chronic etiology that is rooted in an imbalanced homeostasis of both blood lipids and oxidative status. Such disorders are responsible for a long-standing process resulting in the accumulation of modified low-density-lipoprotein-cholesterol (LDL-C) engorged macrophages, or “foam cells”, within the intima of the arterial wall, corresponding to the first step of the atheromatous plaque formation. Various factors, such as chronic inflammation, may lead to plaque rupture until the clot of vessels and rapidly induce heart or brain ischemia [2].

As lipid deposits are a crucial step in foam cell accumulation and the atherosclerotic lesion progress, it appears to be an interesting target to investigate for prevention. The main way the organism is capable of removing the excess lipid deposits and foam cell-derived cholesterol is through reverse cholesterol transport (RCT) mediated by high-density-lipoprotein (HDL-C). This mechanism, based on the cholesterol efflux capacity (CEC), manages the clearance of cholesterol from peripheral tissues, including the arterial wall, and it has recently been established to be a relevant predictor of cardiovascular risk [3,4]. Accordingly, the stimulation of RCT might slow down the formation and the development of atheroma [5,6].

As a first-line strategy in the primary prevention guidelines for CVDs, lifestyle modifications, and namely the adherence to a healthy diet, is of great importance. Thus, the Mediterranean diet has been described as an efficient nutritional approach for the prevention of CVDs, as it has been demonstrated by both epidemiological and experimental studies [7,8]. This dietary pattern is characterized by a high consumption of fresh fruit and vegetables, cereals, fish, olive oil and moderate amounts of red wine. From a nutritional perspective, it has been assumed that a higher and chronic consumption of Mediterranean diet-associated polyphenols, the most abundant bioactive micronutrients within all fruits and vegetables, is reliable in terms of the various health effects ascribed to them [9].

In recent years, numerous in vivo studies, as well as clinical trial interventions, have displayed the beneficial effects of polyphenolic compounds in the primary prevention of CVDs, either associated with drugs, such as statins, or with a controlled diet [10,11]. Beyond their highly-studied antioxidant and anti-inflammatory effects, recent researches have highlighted the modulatory capacity of polyphenols on several cellular signaling pathways of interest, and, of note, on the modulation of cellular lipid metabolism [10]. Although some individual flavonoids have demonstrated preventive effects for atherosclerosis development through their capacity to regulate RCT [12], nevertheless, little is known about potential benefits of a complex blend of polyphenols as it is regularly ingested with the Mediterranean diet.

In this regard, the present research aimed at evaluating the effects of a 12-week supplementation with Oleactiv^®^, a fruit and vegetable-based ingredient rich in various polyphenol compounds extracted from grape, olive and artichoke, in a *Golden Syrian* hamster model with high-fat diet-induced early-stage atherosclerosis.

## 2. Materials and Methods

### 2.1. Test Supplement

Oleactiv^®^, supplied by FYTEXIA (Vendre, France), is obtained by alcohol and water extraction of olive (*Olea europaea* L.), artichoke (*Cynara cardunculus var. scolymus*) and both, white and red grape pomace and seed (*Vitis vinifera* L.). The supplement provides bioactive compounds, especially polyphenols from phenolic acids, flavonoids and the phenylethanoid families. The supplement was analyzed by means of high-performance liquid chromatography (HPLC) using an Agilent HPLC 1260 apparatus (software Openlab CDS chemstation edition, Agilent Technologies, Santa Clara, CA, USA) coupled with a diode array detector. Separation was carried out by mean of a Zorbax Stablebond SB-C18 column (4.6 × 1.5 mm; 5 µm particle size, Agilent Technologies, Santa Clara, CA, USA). To detect different phenolic classes, the analytical method was set at two different wavelengths: 280 nm for flavonoids and phenylethanoids/secoiridoids and 350 nm for phenolic acid compounds. The mobile flow rate was 0.8 mL/min with an injection volume of 25 µL. The solvents used were (A) water, (B) acetic acid (Honeywell, Muskegon, MI, USA)and (C) acetonitrile (Honeywell, Muskegon, MI, USA)and the linear gradient program was set as follows: (a) 99% A and 1% B (0 min); (b) 0 to 15 min linear gradient to 94% A and 6% B; (c) 94% A and 6% B (15 to 30 min); 30 to 50 min linear gradient to 91.1% A, 5.9% B and 3% C; (d) 50 to 60 min linear gradient to 88.2% A, 5.8% B and 6% C; (e) 60 to 80 min linear gradient to 85.3% A, 5.7% B and 9% C; (f) 80 to 120 min linear gradient to 65% A, 5% B and 30% C; (g) 65% A, 5% B and 30% C (120 to 140 min); (h) 100% A (140 to 155 min). Flavonoids, phenylethanoids/secoiridoids and phenolic acid compounds were respectively expressed as catechin, oleuropein and chlorogenic acid equivalents. Catechin and oleuropein standards were purchased from Sigma-Aldrich Co. (St. Louis, MO, USA) and the chlorogenic acid standard from Extrasynthese (Genay, France).

Oleactiv^®^ was administered at a daily dose of 55 mg/kg body weight, which is equivalent to a human equivalent dose (HED) of 450 mg daily. The HED was calculated according to the equation reported by Reagan-Shaw et al. based on both body weight and body surface area [13]. HED (mg/kg) = hamster dose (mg/kg) × (hamster *K_m_*/Human *K_m_*), where the body weight of a human adult was set at 60 kg and the *K_m_* factor, representing the body surface area of either hamster or human was given respectively to be 5 and 37.

### 2.2. Design of the Study

Twenty-one 5-week old male *Golden Syrian* hamsters (*Mesocricetus auratus*), with an average body weight of 85 g, were purchased from Janvier LABS Company (Le Genest-St-Isle, France). The animals were handled in compliance with European Union rules, according to the Committee for Animal Care of University of Montpellier (France), as well as the US Guidelines of the NIH (National Institutes of Health) [14]. After a 1-week acclimation period, animals were randomly assigned to form three groups of similar average body weight. The first group was the negative control and corresponded to animals receiving a standard diet (STD group) (*n* = 7). The second group consisted of animals fed a high fat diet (HFD, Control (CTRL) group) (*n* = 7) which was the positive control model of atherosclerosis induction. The third group comprised animals fed an HFD and supplemented with Oleactiv^®^ (HFD, OLE group) (*n* = 7). Hamsters were grown up in collective cages (either 3 or 4 individuals), housed at 23 ± 1 °C and subjected to a 12 h/12 h light/dark inversed cycle for 12 weeks. The STD group received a balanced diet (total caloric intake: 3885 kcal) corresponding to 958 Kcal from proteins, 2522 kcal from carbohydrates and 405 kcal from lipids. This diet was provided with both corn and colza oil (10.4%). The energy coming from the HFD was 4170 kcal, corresponding to 812 Kcal of proteins, 2440 kcal of carbohydrates and 918 kcal of lipids (mostly hydrogenated coconut oil, 22%). The HFD was also supplemented in cholesterol (0.2%). The details of the diets are described in Table 1. Animals were fed ad libitum, had free access to water and were daily supplemented by oral gavage, with an aqueous solution of OLE (OLE group) or with tap water as vehicle (STD and CTRL groups). At the end of the experimental period, the animals were deprived of food overnight, and after euthanasia, fasting blood samples were collected by cardiac puncture with heparin as anticoagulant. Plasma was isolated from blood by centrifugation at 3000× *g* for 20 min and stored at −80 °C.

### 2.3. Evaluation of Hepatic Steatosis

The liver was excised, weighed, and sectioned for histology. Liver samples were fixed in 10% neutral buffered formaldehyde and paraffin embedded, and 3 µm-thick serial sections were prepared. Sections were deparaffinized and stained with hematoxylin and eosin.

### 2.4. Measurement of Lipid Profile

Plasma total cholesterol (TC), HDL-C, and triglycerides (TG) were determined using commercially available enzymatic kits (CH 200, CH 203, TR 1697 respectively, Randox Laboratories Ltd., Crumlin, UK). HDL-C was measured in the supernatant, after the treatment of plasma with a phosphotungstate reagent, as previously described [15]. Non-HDL-C, which includes LDL-C, was calculated as the difference between TC and HDL-C.

### 2.5. Evaluation of Early Atherosclerosis: Aortic Fatty Streak Area (AFSA)

Following the protocol of Auger et al. [16], the intact aortic arch was firstly perfused with phosphate buffered saline containing 1 mmol/L CaCl_2_ and 15 mmol/L glucose for 5 min, then afterwards with 0.1 mmol/L sodium cacodylate buffer, pH 7.4, containing 2.5 mmol/L CaCl_2_, 2.5% paraformaldehyde and 1.5% glutaraldehyde, in order to fix the vessels. The aorta was carefully dissected and processed as previously described [16], and the lipids were stained with Oil Red O. A microscope equipped with an image acquisition and analysis system (Image J, Scion Corporation, Frederick, MD, USA) was used to analyze the total Oil Red O-stained area for each aortic arch. The area covered by lipid deposits (aortic fatty streak area or AFSA) was calculated as a percentage of the estimated aortic total area (ATA). Equivalent aortic surface areas were compared.

### 2.6. Evaluation of Cholesterol Efflux Capacity (CEC) of Sera

Murine J774 macrophages were radiolabeled with [^3^H]-cholesterol 2 µCi/mL (Perkin Elmer, Waltham, MA, USA) in medium containing 1% fetal calf serum (FCS, Euroclone, Milan, Italy). Following a 24-h labelling period, cells were incubated with 0.2% of bovine serum albumin (BSA, Sigma-Aldrich Corporation, St. Louis, MO, USA), with or without 0.3 mM cpt-cAMP (Sigma-Aldrich Corporation, St. Louis, MO, USA), to up-regulate ABCA1 transporter, for 18 h [17]. A total of 2 µg/mL of ACAT inhibitor (Sandoz 58035, Sigma-Aldrich Corporation, USA) was added during the labelling and equilibration period to prevent the cellular accumulation of cholesteryl ester [18]. Afterward, cells were incubated with 1% (*v*/*v*) hamster plasma for 4 h. Hamster plasma was thawed in ice before addition to cells. The radioactivity of the medium was determined by liquid scintillation counting. Cholesterol efflux was calculated as a percentage of the radioactivity released into the medium over the radioactivity incorporated by cells before the addition of plasma (Time zero). In order to analyze cellular [^3^H]-cholesterol content, cell monolayers were extracted by the addition of 0.6 ml of 2-propanol (VWR International, Radnor, PA, USA). The lipid extracts were dried under a stream of N_2_, re-suspended in toluene (VWR International, USA), and quantified by liquid scintillation counting. The ABCA1-mediated cholesterol efflux was calculated as the percentage efflux from cpt-cAMP-stimulated J774 macrophages minus the percentage efflux from unstimulated J774 cells. ABCA1 upregulation and activation has been certified by treating a parallel set of cells with apoA-I in the presence or absence of cpt-cAMP. In the former, cholesterol efflux was 7.5% ± 0.5%. In the latter: 1.35% ± 0.09%.

### 2.7. Characterization of HDL Particles

Plasma HDL subclasses were characterized according to their surface charge and size by two-dimensional (2D) electrophoresis performed as previously described [19], followed by immunodetection with a commercial polyclonal antibody against murine apoA-I (Meridian Life Science, Memphis, TN, USA) and visualized by enhanced chemiluminescence (GE Healthcare Biosciences, Uppsala, Sweden). Images were acquired with a GS-690 Imaging Densitometer and the Multi-Analyst software, version 1.1 (Bio-Rad Laboratories, Hercules, CA, USA).

### 2.8. Statistical Analysis

Data are shown as means ± SD (standard deviation). Statistical analysis was performed using GraphPad Prism software, version 6.01 (GraphPad Software, La Jolla, CA, USA). Significant differences were established with one-way analysis of variance (ANOVA) and Student’s *t*-test. A *p*-value of ≤0.05 was taken to indicate a significant difference.

## 3. Results

### 3.1. Characterization of the Polyphenolic Profile of the Supplement

Total bioactive content identified in the supplement corresponded to 16.1 g/100 g dry matter. The total flavonoid content from the grape extracts were measured at 4.2 g/100 g; the total phenolic acid content from artichoke extract corresponded to 2.8 g/100 g; and the total phenylethanoid/secoiridoid content from the olive extract was 9.1 g/100 g (Table 2).

### 3.2. Biometric Parameters and Food Intake

As expected, after 12 weeks, body weight of the CTRL group was increased when compared to the STD group (+29%, *p* < 0.0001). Conversely, the body weight of hamsters within the OLE group was not different from the CTRL group (Table 3). A similar trend was observed for the liver. When compared to the STD group, where the liver weight normalized to body weight, within the CTRL group it had significantly increased (+52%, *p* < 0.0001), whereas it remained stable within the OLE group (Table 3). Both the CTRL and OLE groups had an increased food intake by respectively 32% (*p* < 0.0001) and 34% (*p* < 0.0001), when compared to the STD group.

### 3.3. Hepatic Steatosis

**Figure 1:**
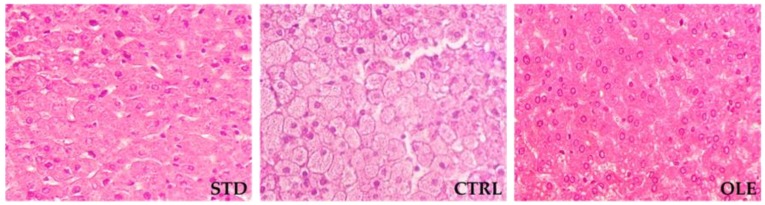
Histological evaluation of hepatic steatosis in standard diet (STD), high-fat diet (CTRL), or Oleactiv^®^ (OLE) hamsters after 12 weeks of supplementation. Representative liver sections (40× magnification) are illustrated.

STD-fed hamsters did not exhibit any histological evidence of hepatic steatosis. In contrast, the CRTL group displayed micro-vesicular steatosis of moderate to severe intensity. The animals within the OLE group, despite having had a similar liver weight to those in the CTRL group, did not develop hepatic steatosis (Figure 1).

### 3.4. Plasma Lipid Profile

**Figure 2:**
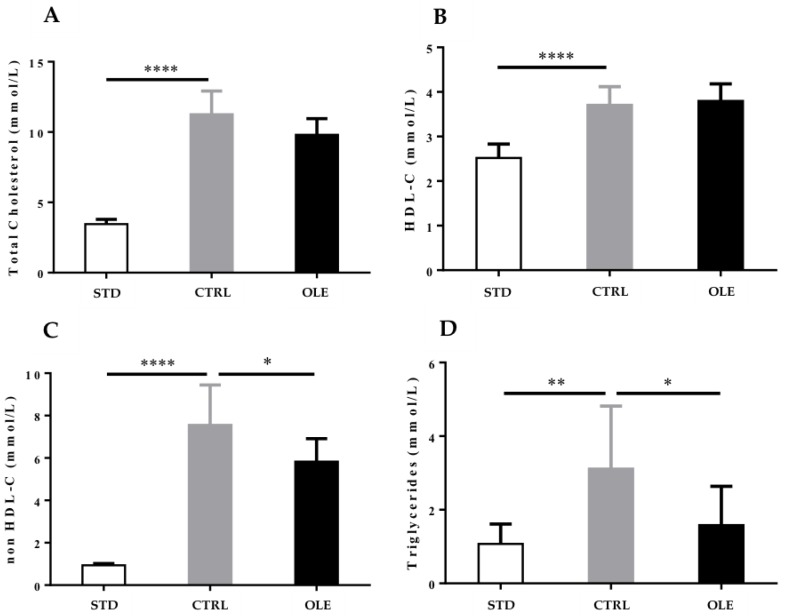
Oleactiv^®^ modulation of hamsters’ lipid profile: analysis of total cholesterol (TC) (**A**) HDL-C; (**B**) triglycerides (TG); (**C**) and non-HDL-C; (**D**) after 12 weeks of supplementation, in hamsters fed with a standard diet (STD group), a high-fat diet (CTRL group), or a high-fat diet and Oleactiv^®^. Statistical analysis were performed using ANOVA one way, with Dunnett’s multiple comparison test. (* *p* < 0.05; ** *p* < 0.01; **** *p* < 0.0001).

Hamsters from the CTRL group developed a significant dyslipidemia related to the intake of the HFD when compared to the STD group. It is noteworthy that the animals displayed a significant increase of TC (+227%, *p* < 0.0001), HDL-C (+47%, *p* < 0.0001), non-HDL-C (+704%, *p* < 0.0001), and TG (+192%, *p* < 0.001). The OLE supplementation induced a non-significant decrease in TC (−13%, *p* = 0.0578), whereas HDL-C stayed stable when compared to the CTRL group. Conversely, a significant reduction of non-HDL-C (−23%, *p* < 0.05) and of TG (−49%, *p* < 0.05) was observed (Figure 2A–D).

### 3.5. Aortic Fatty Streak Area (AFSA)

**Figure 3:**
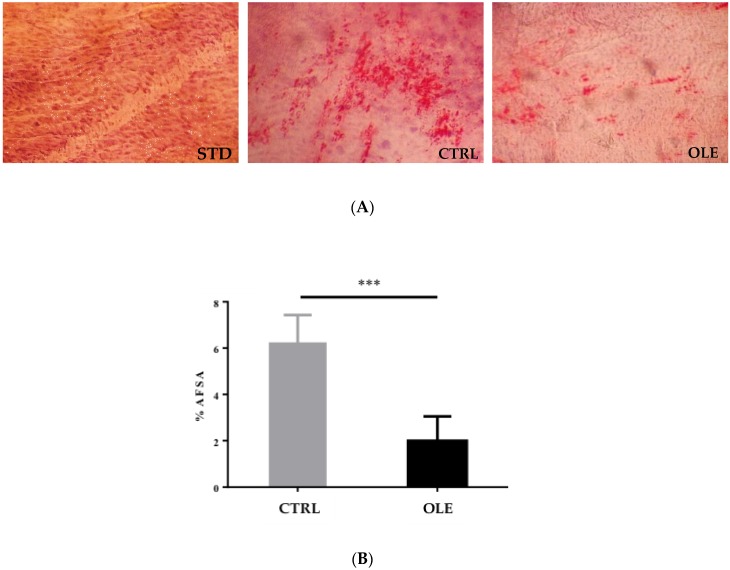
Lipid deposits in the aortic wall of hamsters: Photomicrographs of sections of the surface of the aortic arch stained with Oil Red O (**A**) and aortic fatty streak area (AFSA) to aortic total area (ATA) ratio in hamsters of STD group, CTRL group, and OLE group (**B**). Statistical analysis were performed using the Student’s *T*-test (*** *p* < 0.001).

The HFD induced the formation of fatty streaks in hamsters of the CTRL group. These animals presented a significant fat deposition in the intima of vessels, representing 6.2% of the total area covered by lipid deposits. Interestingly, within the OLE group, a significantly lesser lipid deposit (−69%; *p <* 0.0001) was observed when compared to the CTRL group (Figure 3A,B).

### 3.6. Cholesterol Efflux Capacity of Hamster Plasma

**Figure 4:**
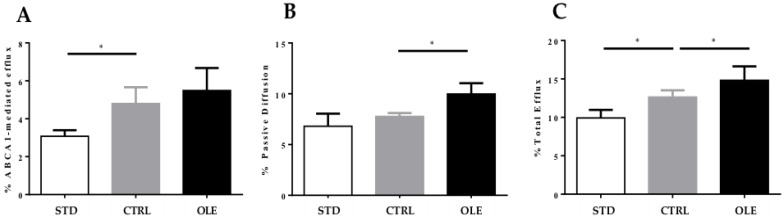
Cholesterol efflux capacity of hamster plasma: evaluation of cholesterol efflux from J774 macrophages incubated with hamster plasma treated with standard diet (STD group), or high-fat diet (CTRL group), or a high-fat diet and with Oleactiv^®^ (OLE group): % ABCA-1-mediated efflux (**A**), % passive diffusion (**B**) and % total efflux (**C**). The experiment was performed in triplicate. Efflux was expressed as counts per minute in medium/counts per minute of time 0 ×100 ± SD. Statistical analysis were performed using ANOVA one way, with Dunnett’s multiple comparison test. * = *p* < 0.05.

When compared with the STD group, the plasma of the hamsters from the CTRL group produced a significant increase in ABCA1-mediated efflux (+55.84%, *p* < 0.05), but did not display any difference in the passive diffusion contribution, corresponding altogether to a significant increase of total CEC (+27.29%, *p* < 0.05). When compared to the CTRL group, the OLE supplementation induced a slight increase of ABCA1 cholesterol efflux by 14.38%, and above all, a significant increase of passive diffusion (+28.44%, *p* < 0.05), corresponding altogether with a significant increase of total CEC (+17.33%, *p* < 0.05) (Figure 4A–C).

### 3.7. HDL Particle Characterization

**Figure 5:**
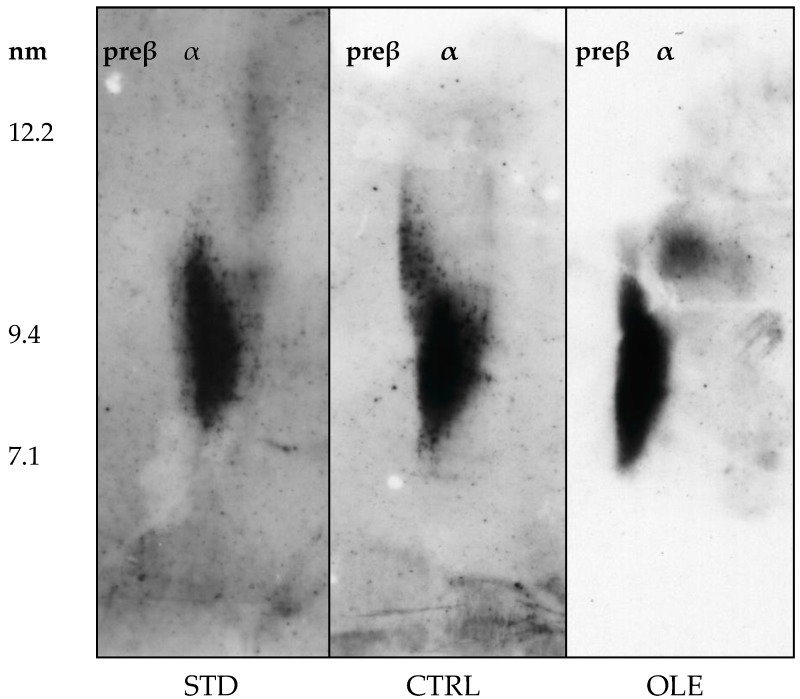
Plasma HDL characterization of hamsters: HDL subclasses were separated by 2D electrophoresis then transferred onto a nitrocellulose membrane, on which lipoproteins were detected with an anti-murine apoA-I antibody. Plasma from four STD, four CTRL and nine OLE were evaluated; images are shown for one representative sample for each group. α, specific sub-population of HDL.

In contrast to those detected in humans and other rodents, HDL from hamsters did not present pre-beta fraction (Figure 5).

When compared to the CTRL group, the high fat diet induced a slight reduction of HDL size that was even more evident in animals treated with OLE supplementation.

## 4. Discussion

In the present research, we evaluated the effects of Oleactiv^®^, a polyphenol-rich ingredient obtained from the extraction of fruit and vegetables commonly consumed within the Mediterranean diet, on atherosclerosis etiology in high-fat fed and hypercholesterolemic hamsters. Animals from the OLE group had normal growth and their livers were comparable to the CTRL group. Moreover, the lack of steatosis signs in the hamster livers of the OLE group strengthened the protection of the OLE supplementation. Interestingly, a 12-week long period of supplementation with OLE revealed an athero-protective aptitude, preventing the high-fat diet-induced formation of atheroma in hamsters. The supplementation reduced fat deposits within the arterial wall—the early stage of the principal mechanism of atherosclerosis development [20]—through the increase of cholesterol uptake from modified lipoproteins and foam cells [21] deposited within the arterial intima.

A substantial body of evidence shows that CEC measurement is a relevant biomarker for the prediction of coronary artery disease. Indeed, many studies have demonstrated that CEC is inversely associated to the occurrence of atherosclerotic-based cardiovascular events [22,23], making this parameter a better predictor of coronary artery disease than the simple measurement of the absolute HDL-C level [3,24]. CEC may therefore be considered an emerging therapeutic target.

From the present research on CEC evaluation, it can be hypothesized that a part of the mechanism reliable for a significantly lower percentage of lipids observed within the aortic wall of OLE animals, could be the effect of increased cholesterol efflux from macrophages within the atheroma towards the HDL-C lipoproteins in the circulation. Such a significant outcome was observed in the CEC of the OLE group, which included the contributions of both passive diffusion and ABCA1-mediated efflux; this is a noteworthy beneficial effect of the supplement in the prevention of cardiovascular events. Besides and although no significant change was observed for the HDL-C concentration of the OLE group, nevertheless the increased CEC activity might rather be associated with a remodeling of these lipoproteins. Indeed, the appearance of smaller HDL is consistent with the observed trend towards improved ABCA1-mediated CEC. As is well established, this transporter preferentially interacts with small-sized HDL [25]. On the other hand, the observed increase of CEC occurring via other mechanisms, measured as passive diffusion and total efflux, can be explained by the contribution of apoB-containing lipoproteins [12,26]. Such a positive effect on CEC might be in part ascribed to the olive polyphenol content in the OLE supplement. Indeed, Berrougui et al. reported evidence from a human intervention; authors showed that the polyphenols contained in olive oil are able to both inhibit the oxidation of HDL-C, and also to maintain HDL-C physicochemical properties, therefore strengthening their aptitude to promoting the RCT process [27]. Moreover, it is noteworthy that grape polyphenols, namely catechin and epicatechin, have also been demonstrated to provide similar antioxidant capacities towards both LDL-C and HDL-C [28], and consequently might, on the one hand, limit the LDL-C-based foam cell production, and, on the other hand, have a positive effect on HDL-C-mediated CEC functionality. Regarding the oxidative-based LDL-C foam cell production, various authors have even reported that polyphenol bioactive compounds from olive oil such as the phenylethanoid, hydroxytyrol, or the secoirodoid, oleuropein, have the ability to differentially express 98 genes in humans (up- and down-regulation), of which some are involved in inflammation or in redox homeostasis [29]. Among the upregulated genes, paraoxonase 1 (PON1) participates in this activity, probably in combination with other HDL-C-associated enzymes [30]. Authors have also reported that flavonoids from grape, namely catechins and procyanidins, were also able to up-regulate PON1 activity and protection against LDL-C oxidation. Besides, Acin et al. [31] reported that the size of aortic atherosclerotic lesions was positively correlated with circulating paraoxonase activity in mice, highlighting the importance of the polyphenol-based regulation of PON1 in postponing the development of atherosclerotic lesions.

Beyond its beneficial effect on CEC, OLE supplementation might also help to improve atherosclerotic risk through different mechanisms. The proposed modification of the HDL quality may affect not only CEC, as directly assessed, but also the cholesterol loading capacity of lipoproteins. Thus, it could be speculated that Oleactiv treatment reduces lipoprotein donor function, resulting in a less pronounced enrichment of cells with cholesterol-derived lipoproteins. Indeed, in the present research, the supplement was demonstrated to significantly modulate the lipid profile of the HFD-fed animals. Animals from the OLE group clearly displayed, after 12 weeks of supplementation, a significant decrease in TG, LDL-C and VLDL-C (expressed as non-HDL-C), as well as a slight decrease in total cholesterol, when compared to the results from the CTRL group. Although the mechanism that underlies such results was not directly evaluated, we might speculate that this hypolipidemic effect could easily be related to the reduction of both lipid intestinal absorption, probably through interaction with lipase activity [32,33], and also to the reduction of hepatic cholesterol synthesis, mediated by polyphenols contained within the supplement [34,35]. Besides, various research teams have also demonstrated the beneficial effects of several other polyphenol compounds on the gene expression of the specific transporters involved in both lipid absorption and in the lipid metabolism in the intestine and liver [36]. Thus, Qin et al. reported from a study conducted in enteric cells that the administration of a cinnamon extract, which is a significant source of caffeic acid derivatives, as in the case of artichoke, was able to inhibit the expression of some genes associated with cholesterol uptake and with lipogenic pathways, but also to facilitate ABCA-1 expression [37]. Moreover, the polyphenol compounds, luteolin and quercetin, that have been identified in artichoke and red grape respectively, have been reported to be able to reduce the expression of the NPC1L1 transporter in the human intestinal cell model, Caco-2, as well as in a HEK cell transfected model that overexpresses NPC1L1 [38]. Regarding the modulation of the hepatic synthesis of cholesterol, several data have demonstrated the capacity of numerous polyphenols, especially from pomegranate, artichoke and grape extracts, to either modulate or even to completely inhibit endogenous cholesterol synthesis [39,40]. In addition, the evidence from the literature suggests that artichoke polyphenols might inhibit cholesterol synthesis directly through their effect on HMG-CoA reductase activity, the key enzyme of cholesterol biosynthesis [41]. Finally, Rouanet and his colleagues confirmed the hypolipidemic and anti-atherosclerotic effects of polyphenolic extracts from grape in hypercholesterolemic hamsters [42].

An interesting aspect of this research was the use of *Golden Syrian* hamsters. Indeed, this animal model is a good representation of cholesterol metabolism and the enzymatic pathways involved in atherosclerotic processes when compared to other rodent models that are naturally resistant to diet-induced atherosclerosis [43]. To study the effect of a food supplement on HFD-induced atherosclerosis, the main of the advantages of this model were that hamsters have a receptor-mediated uptake of LDL cholesterol, cholesteryl ester transfer protein (CETP) activity, hepatic apoB-100 and intestinal apoB-48 secretion that are comparable to humans [44]. These similarities imply a similar behavior in atheroma formation and development. Accordingly, the study of candidates for atherogenesis prevention with this model predicts more reliably than other rodent models the effects on the modulation of both CEC and the lipid profile in humans.

The regular administration of Oleactiv^®^, by reducing plasmatic lipids and improving CEC, could represent a promising approach for the prevention of atherosclerotic development in humans with light or moderate hyperlipidaemia, but who are otherwise healthy. Further investigations should be conducted to confirm the bioavailability of polyphenols from the supplement and identify which metabolites are involved in the beneficial effects reported so far.

## Figures and Tables

**Table 1:** Composition of the standard and high fat diet administered for 12 weeks to hamsters.

	Standard Diet		High-Fat Diet	
Ingredients	%	kcal/kg Diet	%	kcal/kg Diet
Casein	23.60	944	20.00	800
L-Methionin	0.35	14	3.0	12
Corn starch	30.00	1200	39.30	1572
Maltodextrin	3.0	120	5.3	212
Sucrose	29.05	1162	15.40	616
Cellulose	5.0	0	5.0	0
Vegetables oil (corn/soy, 1:1)	4.5	405	0.0	0
Hydrogenated coconut oil	0.0	0	10.00	900
Cholesterol	0.0	0	0.2	18
Minerals mix	3.5	0	3.5	0
Vitamins mix	1.0	40	1.0	40
Total	100	3885	100	4170

**Table 2:** Characterization of the phenolic compounds identified in the Oleactiv^®^ supplement. Rt, retention time.

Compounds	Rt (min)	Λ Max (nm)	Content (g/100 g)
Gallic acid	5.4	272	0.14 ± 0.11
Hydroxytyrosol	12.3	280	2.93 ± 0.35
Procyanidin B1	16.6	278	0.59 ± 0.09
Procyanidin-like	17.3	280	0.38 ± 0.06
Catechin	19.0	280	1.39 ± 0.39
Chlorogenic acid	24.4	300; 326	0.80 ± 0.07
Cryptochlorogenic acid	27.5	300; 326	0.54 ± 0.04
Epicatechin	33.9	278	1.01 ± 0.41
Flavanol-like	36.9	278	0.73 ± 0.13
Cynarin	43.5	302; 318	0.45 ± 0.07
Cynarosid	87.6	348	0.99 ± 0.19
Oleuropein	102.4	281	5.02 ± 0.18
Oleuropein-like	107.4	281	1.13 ± 0.06

**Table 3:** Biometric parameters and food intake.

	STD Group	CTRL Group	OLE Group
**Body Weight (g)**	99.95 ± 15.11	128.73 ± 7.09 ****	125.01 ± 5.40 (ns)
**Liver Weight (g)**	3.74 ± 0.67	7.34 ± 0.85 ****	7.22 ± 0.76 (ns)
**% Liver/Weight**	3.74 ± 0.32	5.70 ± 0.57 ****	5.77 ± 0.54 (ns)
**Food intake (g/day)**	4.57 ± 0.59	6.04 ± 1.07 ****	6.14 ± 1.26 (ns)

Clinical parameters measured after 12 weeks of supplementation of hamsters of the STD group (fed with standard diet), the CTRL group (fed with high-fat diet), and OLE group (fed with high-fat diet and supplemented with OLE). Statistical analysis were performed using ANOVA one way, with Dunnett’s multiple comparison test. **** *p <* 0.0001 compared with STD group and ns = not significant compared to CTRL group.
